# Inter-University Consortium for Political and Social Research (ICPSR)

**DOI:** 10.5195/jmla.2017.120

**Published:** 2017-01

**Authors:** Stephanie M. Swanberg

## PURPOSE AND DESCRIPTION

The mission of the Inter-University Consortium for Political and Social Research (ICPSR) is to “[advance] and [expand] social and behavioral research, acting as a global leader in data stewardship and providing rich data resources and responsive educational opportunities for present and future generations” [1]. The ICPSR was originally founded in 1962 at the University of Michigan as the Inter-University Consortium for Political Research to collect and share data from the American National Election Studies, more commonly known as the National Election Study [2]. It has grown to include data in many disciplines, including medicine, the health sciences, and health professions education. Topics covered include health care access, health care costs, health education, health insurance, Medicaid, Medicare, medical evaluation, mental health, medical education, and more. In addition to maintaining this data archive, the ICPSR develops and sponsors educational activities in research design, statistics, and methodology and supports research in data science and curation.

ICPSR primarily targets faculty, researchers, and students at academic institutions but would be of interest and value to anyone searching for existing research data to analyze in a variety of disciplines and topics. ICPSR could be especially beneficial to medical students, residents, and other health professions students who are required to complete an independent research project as part of their training and who are interested in analyzing existing data.

As of July 2016, ICPSR contains 9,676 datasets, about 7,700 of which are publicly accessible. As such, many of the datasets found in ICPSR, particularly those that originate from federal agencies or are federally funded, may also be available in other online data repositories, such as Data.gov. Nearly all datasets (a little over 7,900) originate from studies conducted in the United States, but international data—which may be difficult to locate elsewhere—is also included in ICPSR.

## SEARCHING THE INTER-UNIVERSITY CONSORTIUM FOR POLITICAL AND SOCIAL RESEARCH (ICPSR) DATA ARCHIVE

Locating datasets in ICPSR is very straightforward. From the home page, you can use the single search box and enter a few keywords to start your search. When a keyword search is conducted, the results page is divided into three tabs—Studies, Variables, and Publications—with results sorted by relevance. Studies lists the name of retrieved studies or datasets, Variables lists studies that measured variables with your keywords, and Publications includes full citations for published articles based on data archived in ICPSR.

Each tab features a number of filters you can use to further narrow your search. For example, filters in the Studies tab include geography, data format, type of analysis (quantitative, qualitative), time period, and restriction type (publicly accessible or restricted). Variables and Publications have their own unique sets of filters and results sort options. In Studies, you may also notice an openICPSR icon next to some entries. This indicates that the dataset is available in ICPSR’s new open data repository, openICPSR. openICPSR allows investigators to rapidly self-archive their data in current form without the rigorous data curation process found in ICPSR’s traditional deposit system, discussed below. Currently, openICPSR includes only 147 datasets.

In addition to keyword searching, ICPSR has extensive browse capabilities. From the ICPSR home page, click on the “Find & Analyze Data” heading above the search box to view the different browsing options: topic, series (groups of datasets under the same longitudinal study), geographic location, investigator (individuals or organizations), international data, thematic collections, or all datasets. ICPSR has collaborated with a number of institutions to organize the thematic collections, which include arts and culture, criminal justice, demographics, disability, teaching, race, and health/mental health. For those who are practicing, teaching, or learning medicine, browsing these themed collections is a good starting point for identifying the types of health-related data archived in ICPSR.

Each dataset has its own web page with extensive metadata including access notes (publicly available or members only), study variables, links to download the data files, study description, funding information, and export information. Data files in ICPSR are carefully examined for completeness and quality and are archived in multiple formats to ensure maximum accessibility, including SAS Transport, SPSS Portable, Stata data files, R programming language files, ASCII encoding standard files, or tab-delimited files for use in Excel. Each entry also includes an accompanying codebook in portable document format (PDF) for interpreting the data. Availability of quantitative data in multiple file formats is a hallmark and major benefit of searching ICPSR over other data repositories, as data are typically only available in a single file format from other resources. To download the data—even publicly accessible data—you will need to create a free MyData account in ICPSR. ICPSR allows you to either create a new account or use an existing Google, Facebook, LinkedIn, or ORCID account to sign up.

## DEPOSITING DATA TO THE ICPSR DATA ARCHIVE

In addition to searching for existing datasets, ICPSR encourages investigators from any institution to deposit data to the ICPSR archive for free. ICPSR requires each submission to include all data and documentation needed to understand and interpret the dataset. They particularly encourage depositing SAS Transport, SPSS Portable, or Stata data files, but also accept the other file formats listed above. Depositors need to maintain respondent confidentiality by removing unnecessary respondent identifiers and protecting confidential data that will be included as part of datasets. ICPSR has several approaches for preserving confidential data, including restricting access to member institutions for some datasets, tracking use purposes for datasets containing confidential data through a data request form, requiring use of specific statistical software for analysis, and storing physical data at the ICPSR’s building in Ann Arbor, Michigan, where it can be accessed only in person. Questions regarding confidentiality are included as part of the deposit application process.

Once the files are deposited, ICPSR reviews them thoroughly for completeness, confidentiality, and quality; generates metadata for the dataset to be published in the archive; and creates additional file types for accessibility. Hence, data might not be officially published for a few months after submission. Investigators who wish to publish their data immediately without any revisions from ICPSR can deposit their data in openICPSR. This service is free to investigators at member institutions; nonmembers are required to pay a deposit fee of $600.

In addition to the data archive, the ICPSR website also includes a teaching and learning section with resources for instructors and undergraduate students. Resources include learning guides, online exercises or modules, and an assignment builder for integrating data or data analysis into courses. These materials could be suggested to faculty for use in undergraduate health professions programs such as nursing and are appropriate for higher-level students in medical education.

ICPSR is truly a leader in data curation and archiving, providing higher-quality data, metadata, and documentation than you can find in other data repositories at this time. For those at research-intensive institutions, an institutional membership with ICPSR would be well worth the cost. Even if institutions cannot afford the cost of membership, ICPSR can still be a recommended resource for any investigator, regardless of setting or research experience.
